# Developing a Drug Price Reference Index in the Philippines

**DOI:** 10.1186/2052-3211-8-S1-P7

**Published:** 2015-10-05

**Authors:** Manuel A Haasis, Anna M Guerrero, Marinette J Ladioray

**Affiliations:** 1 National Center for Pharmaceutical Access and Management (NCPAM), Department of Health, Tayuman, Manila, 1003, Philippines

## Objectives

To develop a method of setting a Drug Price Reference Index (DPRI) in the Philippines to ensure good value for money in the procurement and reimbursement of essential medicines.

## Methods

A database of prevailing drug procurement prices was created from actual purchase orders submitted in 2013 by government procuring entities in the Philippines. The database includes information on the unit cost, volumes of procurement, source/supplier/manufacturer, brand, mode of procurement and location of the hospital for each formulation and strength of all drugs in the National Formulary. Multivariate regression analyses were performed for commonly sourced essential drugs exploring possible determinants of drug costs, which include quantities procured and hospital bed capacity. Further cost comparisons were made for other potential determinants such as mode of procurement, supplier/manufacturer and distance of distribution.

## Results

Price data were analyzed for 20 drug products with the highest share of procurement in terms of volume and value. Extreme wide variations in unit costs were consistently observed for all drugs analyzed. The price differentials, i.e. high/low ratio, were found to be up to 60 times when comparing the highest with the lowest priced drugs. The variations in prices were not associated with volumes procured, distance of distribution and hospital bed capacity. Suppliers were also observed to charge different prices for the same brands to different public hospitals, indicating information asymmetry on reasonable prices of drugs.

## Conslusion

Based on the observed wide variations in drug procurement prices in the Philippines, setting the DPRI at the median value for most drugs was found to be an appropriate method to set ceiling prices for public sector procurement. For monopolized pharmaceutical products, other methods may be more appropriate such as value-based pricing, price negotiations and external reference pricing to relevant countries.

